# Application of an original improved U-pillow in patients with prone position ventilation: A comparative retrospective observational study

**DOI:** 10.1097/MD.0000000000048204

**Published:** 2026-04-10

**Authors:** Xuekui Wang, Lisang Fu, Yanbin Ye, Huilin Chen, Yan Wang, Biying Huang, Meifen Chen, Meixian Chen, Limei Zhu, Xiangwu Huang, Chenxing Jian

**Affiliations:** a Anorectal Surgery Department, Affiliated Hospital of Putian University, Putian, China; b Medical School, Putian University, Putian, China; c Nursing Department, Affiliated hospital of Putian University, Putian, China.

**Keywords:** nursing satisfaction, nursing workload, original modified U-shaped pillow, pressure injury, prone position ventilation

## Abstract

Patients in the prone position in the intensive care unit are at risk of facial skin failure, a concern that has traditionally been managed through frequent repositioning, often necessitating considerable effort from healthcare practitioners. This study aims to evaluate the effectiveness of an original improved U-shaped pillows in preventing pressure injuries to the head and face of patients undergoing prone ventilation. A retrospective analysis was conducted on data from 61 patients who underwent prone positioning ventilation. The study group consisted of 31 patients who used the improved U-shaped pillow, which features 7 independent air chambers designed for dynamic segmental pressure regulation. The control group was composed of 30 patients who underwent decompression using traditional integrated U-shaped sponge pillows, which lack pressure-adjustment capabilities. There were no significant differences between the 2 groups at baseline. While the intergroup difference in facial pressure injury incidence was not statistically significant (6.5% vs 23.3%, *P* = .081) and no significant benefit was detected in the univariate analysis (*P* = .080), a significant advantage of the new pillow emerged after adjusting for potential confounders. In the multivariate regression analysis, the study group demonstrated a significantly reduced incidence of facial pressure injuries compared to the control group (*P* = .048). A comparison of the average number of head position changes per hour between the 2 patient groups during prone positioning indicated that the study group demonstrated significantly better performance than the control group. This difference was statistically significant (0.55 ± 0.10 vs 0.68 ± 0.15, *P* <.001). The satisfaction level among nurses in the study group was significantly higher than that in the control group (3.61 ± 0.69 vs 3.04 ± 0.69, *P* = .003). A statistically significant positive correlation was identified between body mass index and pressure injury incidence (*P* = .004). The application of the original improved U-shaped pillow was associated with a reduced incidence of craniofacial pressure injuries in prone-ventilated patients after adjustment for confounders, while also mitigating the clinical nursing burden and improving job satisfaction among healthcare staff.

## 1. Introduction

Prone positioning ventilation is a commonly employed strategy to enhance oxygenation in individuals afflicted with acute respiratory distress syndrome (ARDS).^[1]^ Studies have demonstrated the effectiveness of intermittent prone positioning in lowering mortality rates among patients with an oxygenation index below 150 mmHg.^[2,3]^ Consequently, the utilization of prone positioning ventilation has gained widespread acceptance in the care of critically ill patients in recent years. However, a significant complication associated with this practice, pressure ulcers, has yet to receive adequate attention.^[4]^ The face poses a particularly vulnerable site for pressure injuries in patients undergoing prone positioning ventilation, with areas such as the forehead, cheeks, eyes, ears, mouth, and nose being at heightened risk.^[5]^ Currently, preventative measures for pressure ulcers in patients undergoing prone positioning ventilation are still in the preliminary stages of development and lack standardized protocols.

**Figure 1.**
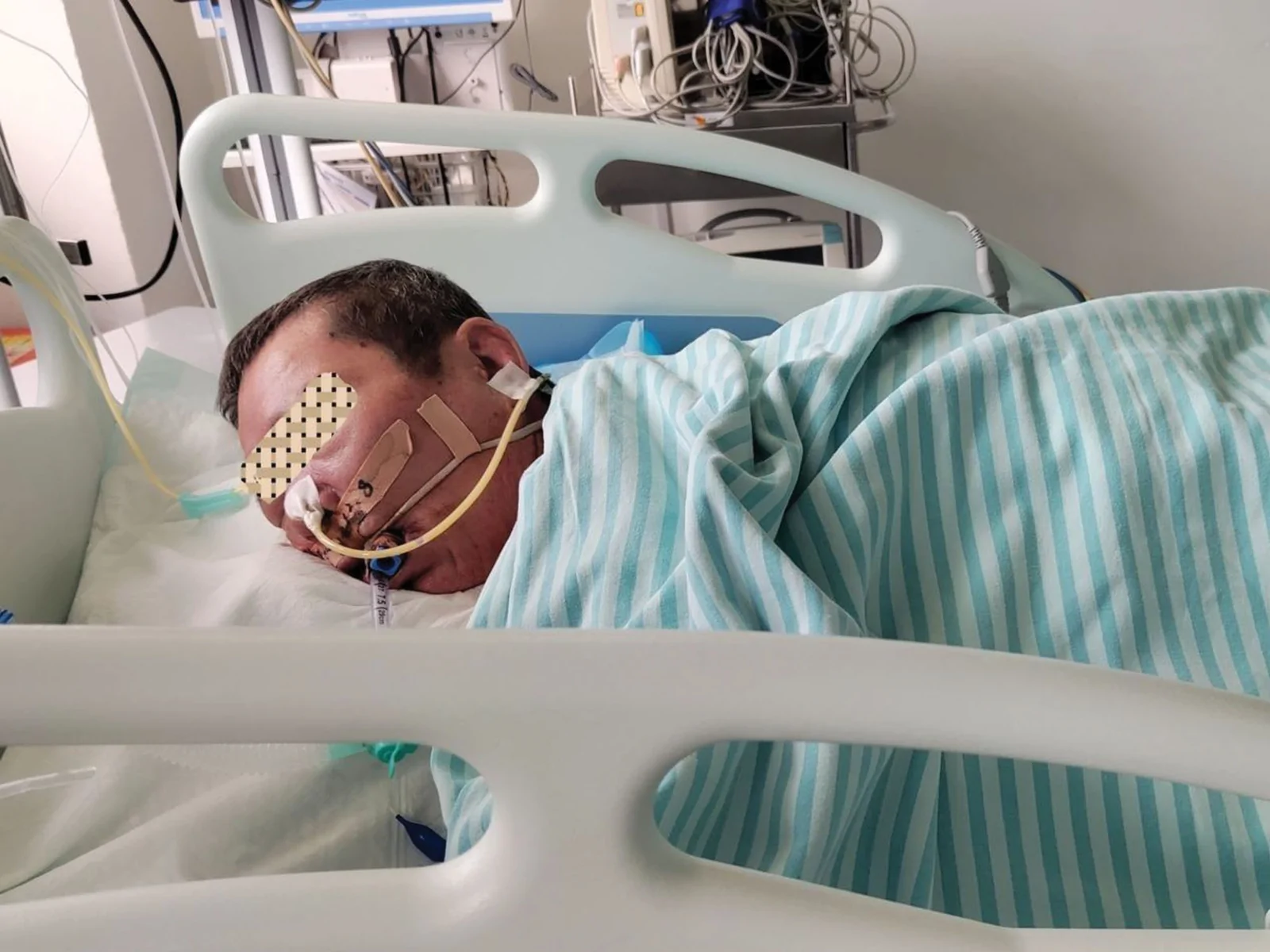
Patients undergoing prone positioning ventilation.

**Figure 2.**
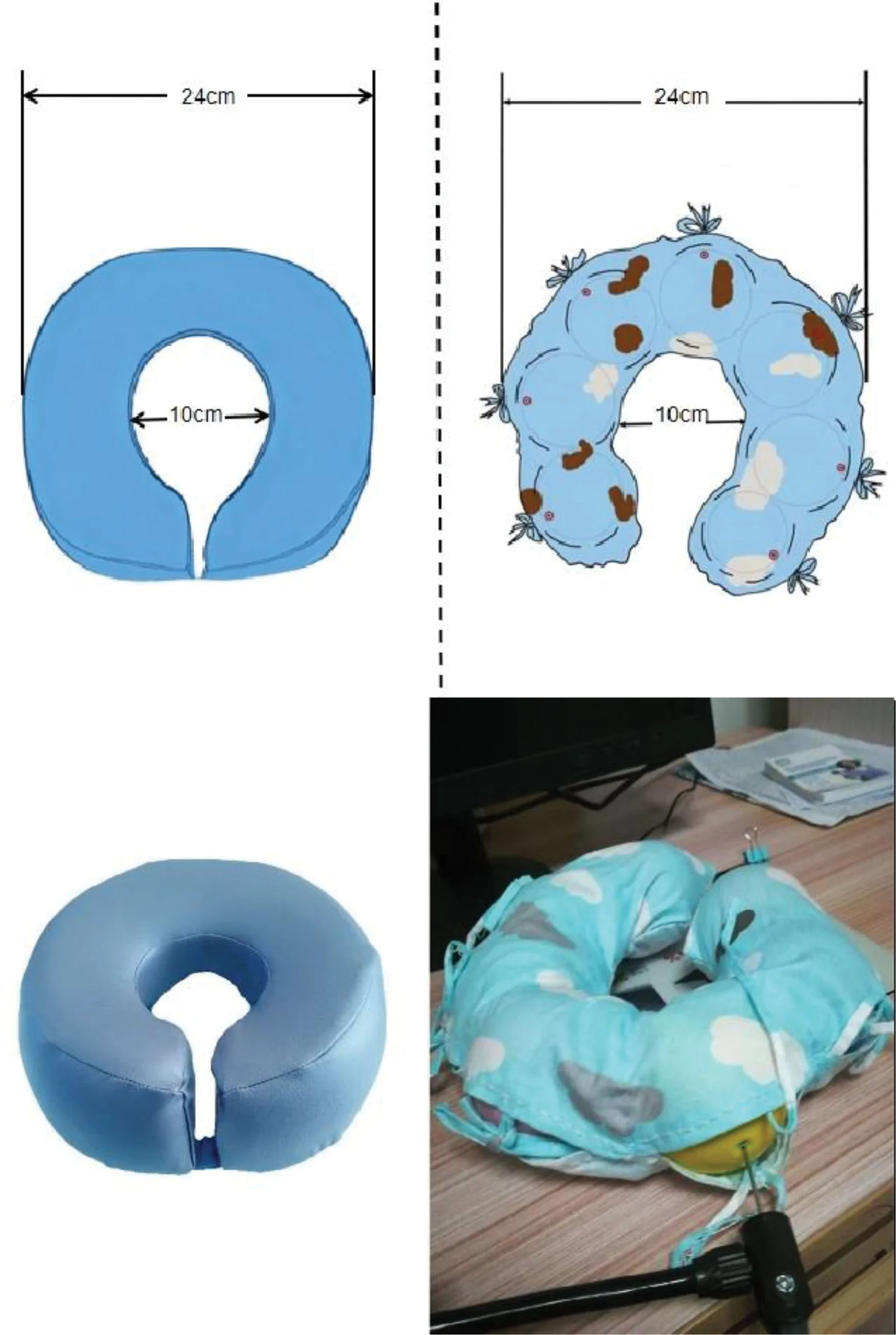
U-shaped pillow: traditional pillow (left), improved pillow (right).

Traditionally, U-shaped pillows and soft dressings are commonly employed to immobilize the head and prevent pressure injuries in patients ventilated in the prone position (Fig. 1). The traditional U-shaped pillow was defined as a monolithic, single-unit cushion constructed from a solid block of foam. Its dimensions featured an outer diameter of 24 cm, an inner diameter of 10 cm, and a consistent height of 7 cm, forming a contiguous support surface (Fig. 2). While these methods aim to mitigate the risk of pressure ulcers, they may inadvertently contribute to their development due to the challenge of easily adjusting pressure points.^[6]^ Hence, we have developed a modified U-shaped pillow designed to not only secure the patient’s head in an optimal position for tracheal intubation but also enhance local tissue perfusion by enabling adjustments to the positioning and duration of pressure on the head and face. This innovation was designed to reduce the incidence of head and facial pressure injuries in patients undergoing prone ventilation while alleviating the workload of nursing staff.

## 2. Methods

Continuous sampling was employed in this study. A total of 61 eligible patients were enrolled from the intensive care unit of the Affiliated Hospital of Putian University between January 1, 2019, and August 30, 2022. The study group consisted of 31 patients utilizing the modified U-shaped pillows, while the control group included 30 patients using conventional U-shaped pillows. The intervention continued until the patient’s condition no longer necessitated prone ventilation or death. This study aims to evaluate the effectiveness of an original modified U-shaped pillow in preventing pressure injuries to the head and face of patients undergoing prone ventilation.

### 2.1. Inclusion criteria and exclusion criteria

Inclusion criteria patients meeting the following criteria were included in this study: undergoing prone positioning ventilation; duration of each prone positioning ventilation session meeting or exceeding 12 hours; receiving invasive mechanical ventilation; oxygenation index ranging from ≤100 to 300 mm Hg and PEEP exceeding 10 cmH2O; RASS sedation assessment tool^[7]^ scores falling within the range of −3 to −4 points.

Exclusion criteria: Patients were excluded from the study if they met any of the following criteria: presence of head and face trauma; Braden risk assessment tool^[8]^ score of ≤12 points; data dropout; ineligibility for prone ventilation due to conditions such as spinal or rib fractures, hemodynamic instability, among others.

### 2.2. Intervention

Modified U-shaped Pillow The modified U-shaped pillow was designed as a circular ring with an inner diameter of approximately 10 cm and an outer diameter of around 24 cm. It is segmented into 7 small compartments (Fig. 2). Each elastic sphere within the pillow is equipped with an air valve for adjusting the elasticity, thereby allowing customization of the pressure exerted on the head and face. The distinctions between the traditional and the improved U-shaped pillow are detailed in Table 1.

**Table 1:** Comparison of traditional U-shaped pillow versus improved U-shaped pillow.

Item	Traditional U-shaped pillow	Improved U-shaped pillow
Shape	U-shaped	U-shaped
Dimensions	Outer diameter: 24 cm, inner diameter: 10 cm, height: 7 cm	Outer diameter: 24 cm, inner diameter: 10 cm, height: 7 cm
Internal structure/compartments	With compartments (7 independent air cells)	Without compartments (monolithic structure)
Filling material	7 individual inflatable air cells (chambers)	A single block of foam
Pressure adjustability	Yes	No
Pressure-adjustment range	Adjustable by modulating the inflation level of individual air cells to alter pressure distribution.	Not applicable (N/A)
Method for adjusting pressure areas	By individually adjusting the pressure in the 7 air cells to dynamically shift pressure areas on the patient’s head and face.	Nonadjustable. Pressure distribution is determined solely by the static shape and density of the foam.

Nursing Methods Both groups of patients had their heads elevated by approximately 20° and received identical routine nursing interventions. A standard repositioning schedule was implemented for patients in both groups, with nurses turning them every 2 hours. This frequency was increased if non-blanchableerythema or skin breakdown was observed on any localized area of the skin. A cyclical pressure-alternation protocol was implemented for the intervention group. During the first hour, the odd-numbered air cells (1, 3, 5, 7) were inflated to a pressure of 2.5 to 3.0 Psi (Pounds per square inch) – a range referenced from clinically established pressures used in alternating-pressure air mattresses – while the even-numbered cells (2, 4, 6) were set below 1.0 Psi. In the second hour, these pressures were reversed: the odd-numbered cells were reduced to below 1.0 Psi, and the even-numbered cells were increased to2.5 to 3.0 Psi. This hourly alternation cycle was continued to systematically shift pressure points. Throughout the process, nurses made fine adjustments based on clinical assessment, ensuring that a finger could be comfortably inserted between the patient’s head/face and the pillow surface, indicating appropriate offloading. The pressure within each air cell was monitored and adjusted using a specialized pressure manometer. A specialized team led by an International Wound/Ostomy Care Nurse was responsible for assessing tissue pressure and determining the need for adjustments to head repositioning frequency. The frequency was increased when pressure injuries beyond Stage I were observed. The patients in this study were all under anesthesia and sedation, making blinding of patients unnecessary. However, blinding could not be applied to the medical staff; therefore, no blinding was conducted in this study.

### 2.3. Outcome measures

#### 2.3.1. Pressure injury occurrence

In accordance with the detailed classification of pressure injuries established by the National Pressure Ulcer Advisory Panel in 2019, pressure injuries are categorized into stages I, II, III, IV, non-staging, and deep tissue injury.^[9]^ The severity of pressure ulcers was assessed using a 5-point Likert scale, with stages I to IV assigned scores ranging from 1 to 4 points and non-staging and deep tissue injuries receiving a score of 5 points.

#### 2.3.2. Nursing single item workload

The head care workload is defined as the average number of head position changes performed per hour during the patient’s prone positioning ventilation period.

#### 2.3.3. Nurse satisfaction survey

A self-designed questionnaire was administered to nurses involved in the care of patients undergoing prone ventilation. The questionnaire was distributed to nurses via WeChat and consisted of 2 sections: general Information: This section included demographic details such as the nurse’s gender, professional title, age, and experience in caring for patients in prone position ventilation. Nurse Satisfaction Survey: This section primarily assessed the operational convenience of the 2 different nursing methods employed. Satisfaction was evaluated using a 5-point Likert rating system, where a score of 1 indicated “very dissatisfied” and a score of 5 indicated “very satisfied.” Prior to administering the questionnaire, a thorough explanation of its purpose, guidelines, and content was provided to the respondents. The questionnaire demonstrated a high retest correlation coefficient (*r* ≈ 0.93). Furthermore, 6 nursing experts with professional designations of deputy chief nurse or higher evaluated the questionnaire’s content validity, confirming a content validity index of 1.

### 2.4. Statistical analysis

All statistical analyses were performed using SPSS (Version 25.0; IBM Corp, Armonk). Continuous data with a normal distribution are presented as mean ± standard deviation and were compared using independent-sample *t*-tests. Categorical data are expressed as numbers (percentages) and were analyzed using the chi-square test or Fisher exact test, as appropriate. To identify independent risk factors for the development of facial pressure injuries, a binary logistic regression analysis was planned a priori. The regression model included key variables such as BMI, age, and duration of prone ventilation. The results are reported as odds ratios with corresponding 95% confidence intervals. To ensure model clarity and avoid confounding, the different intervention groups (traditional vs improved U-shaped pillow) were analyzed separately rather than combined into a single model. A 2-sided *P*-value of <.05 was considered statistically significant.

### 2.5. Ethical approval

This retrospective study was conducted in accordance with the ethical standards of the Affiliated Hospital of Putian University and was approved by its Institutional Ethics Committee (Approval No. 202490). The requirement for informed consent was waived due to the use of anonymized retrospective data. The study was registered with the Chinese Clinical Trial Registry (Registration No. ChiCTR2400089167).

## 3. Result

Demographic details of the patients are presented in Table 2. There were no significant differences between the 2 groups at baseline.

**Table 2:** Characteristics of the 2 groups of patients.

variable	Study group(n = 31)	Control Group (n = 30)	*t*/χ^2^	*P*-value
Age	57.0 ± 8.4	57.5 ± 6.7	0.290	.773
Gender				
Male	26(83.9)	25 (83.3)	–	1.000*
Female	5 (16.1)	5 (16.7)	–	
BMI	22.1 ± 2.5	22.8 ± 3.5	0.948	.347
Classification of diseases				
ARDS	20 (64.5)	18 (60.0)	1.630	.956*
Sudden cardiac arrest	6 (19.4)	5 (16.7)		
Lung cancer	0 (0.0)	1 (3.3)		
MODS	4 (12.9)	4 (13.3)		
H1N1 pneumonia	1 (3.2)	2 (6.7)		
Duration of prone position ventilation (h)	28.3 ± 5.2	28.7 ± 4.7	0.349	.729
RASS scores				
−3	14(45.2)	15(50.0)	0.143	.705
−4	17(54.8)	15(50.0)		
Braden scores	13.7 ± 0.9	13.8 ± 0.8	0.113	.911

* Fisher exact test.

ARDS = acute respiratory distress syndrome, BMI = body mass index, MODS = multiple organ dysfunction syndrome.

### 3.1. Pressure ulcer occurrence

Table 3 presents a comparison of the occurrence of facial pressure injuries between the 2 patient groups. In the control group, which comprised 30 patients, 7 individuals developed pressure ulcers of varying degrees on different areas of the head and face, including 4 cases of stage I pressure injury and 3 cases of stage II pressure injury. Among the 31 patients in the study group, only 2 developed stage I pressure sores on the cheeks. While the between-group comparison did not reach statistical significance (*P* = .81), the study group demonstrated a nonsignificant trend toward reduced incidence of the occurrence of facial pressure injuries compared to the control group.

**Table 3:** Comparison of head and face pressure injury between the 2 groups.

	Incidence of pressure injuries (%)	pressure injuries stage II, n	pressure injuries stage I, n	*P*-value
Study group	6.5	0/31	2/31	.081*
Control group	23.3	3/31	4/31	

* Fisher exact test.

### 3.2. Risk factors for pressure injuries

Initial analysis using Fisher exact test did not show a significant advantage for the control group. Therefore, we conducted further regression analysis. While univariate regression analysis still did not reveal a significant advantage for the new pillow, multivariate regression analysis demonstrated a significant advantage. A statistically significant positive correlation was identified between BMI and pressure injury incidence (*P* = .004). Regarding the duration of prone ventilation, Fisher test and univariate regression analysis revealed significant associations, but this association disappeared in the multivariate regression analysis. Demographic variables including age, gender, and primary disease diagnosis showed no significant associations with pressure injury development, as presented in Table 4.

**Table 4:** Analysis of risk factors for pressure injuries.

	Pressure injuries (n = 9)	No pressure injuries n = 52)	*t*/χ^2^	*P*-value	Univariate regression analysis		Multivariate regression analysis	
					OR (95% CI)	*P*-value	OR (95% CI)	*P*-value
Group								
Control	7 (77.8)	23 (44.2)	–	.081*	0.227 (0.043–1.197)	.080	0.117 (0.014–0.982)	.048
Study	2 (22.2)	29 (55.8)	–					
BMI	19.9 ± 3.2	22.9 ± 2.8	2.977	.004	0.682 (0.509–0.915)	.011	0.728 (0.544–0.972)	.031
Age	61.1 ± 8.4	56.6 ± 7.3	1.690	.096	1.095 (0.984–1.218)	.096	1.100 (0.960–1.262)	.171
Duration of prone position ventilation (h)	32.33 ± 5.85	27.81 ± 4.47	2.679	.010	1.218 (1.307–1.430)	.016	1.203 (0.971–1.490)	.091
Gender								
Male	7 (77.8)	44 (84.6)	–	0.633*	–	–	–	–
Female	2 (22.2)	8(15.4)	–		–	–	–	–
Classification of diseases								
ARDS	7 (77.8)	31 (59.6)	5.494	.212*	–	–	–	–
Sudden cardiac arrest	1 (11.1)	10 (19.2)			–	–	–	–
Lung cancer	1 (11.1)	0 (0.00)			–	–	–	–
MODS	0 (0.0)	8 (15.4)			–	–	–	–
H1N1 pneumonia	0 (0.0)	3 (5.8)	–	–	–	–	–	–

* Fisher exact test.

### 3.3. Nursing single item workload

A comparison of the average number of head position changes per hour between the 2 patient groups during prone positioning indicated that the study group demonstrated significantly better performance than the control group. This difference was statistically significant (*P* <.001), as shown in Table 5.

**Table 5:** Single nursing workload and nurses’ satisfaction were compared between the 2 groups.

	Single nursing workload (times of changes in head position)	Nurses’ satisfaction
Study group	0.55 ± 0.10	3.61 ± 0.69
Control group	0.68 ± 0.15	3.04 ± 0.69
*t*	3.703	3.103
*P*-value	<.001	.003

### 3.4. Nurse satisfaction survey

A total of 29 questionnaires were distributed, of which 1 invalid response was excluded due to the respondent’s lack of involvement in the nursing of patients in the prone position. Consequently, 28 valid questionnaires were collected. The demographic data revealed that 2 respondents were male (7.14%) and 26 were female (92.86%). Among the respondents, 6 held primary professional titles, representing 21.43%, while 22 (78.57%) held intermediate professional titles. The results indicated that the satisfaction level among nurses in the study group was significantly higher than that in the control group. This difference was statistically significant (*P* = .003), as illustrated in Table 5.

## 4. Discussion

Demographic variables including age, gender, and primary disease diagnosis demonstrated no significant associations with pressure injury development. The significant advantage of the research group was not observed in the Fisher test or univariate analysis but emerged in the multivariate regression analysis. We believe this result aligns more closely with clinical reality, suggesting that this pillow achieves better clinical effects when multiple factors interact. Our research indicates that the new pillow has significant advantages over the traditional pillow. Higher BMI was also associated with increased risk. Regarding the duration of prone ventilation, significant differences found in Fisher test and univariate analysis were not sustained in the multivariate model, which might be related to timely assessment and repositioning by nurses in clinical practice.

Prone positioning ventilation is a beneficial approach that enhances lung gas exchange, increases arterial oxygen pressure, and reduces mortality rates in patients with acute respiratory distress syndrome), making it a prevalent modality in clinical practice. As nurses are primarily responsible for implementing prone ventilation, they play a crucial role in anticipating and preventing potential complications associated with this technique.^[10]^ Pressure injuries are a common complication observed during prone ventilation, with stress-induced tissue deformation identified as a significant etiological factor contributing to the development of pressure ulcers.^[11]^

In this study, pressure sores were observed concurrently on both the cheeks and forehead in 4 cases, exclusively on the cheeks in 2 cases, on the nasal tip in 1 case, and on the mandible in 2 cases. These findings identify the cheeks, forehead, nasal tip, and mandible as primary sites for pressure injuries in patients undergoing prone positioning ventilation.

Compared to other critically ill patients, individuals diagnosed with ARDS exhibit an increased susceptibility to developing pressure ulcers. The prevalence of stress hyperglycemia in ARDS patients compromises the effective utilization of glucose during hyperglycemic episodes, resulting in elevated consumption of proteins and fats. Notably, malnutrition among ARDS patients can reach as high as 74%,^[12]^ This malnutrition frequently leads to decreased skin resilience, rendering patients more vulnerable to damage and infection, thereby heightening their risk for pressure injuries. Additionally, variations in nutritional status contribute to a significant discrepancy in body mass index (BMI) between patients with and without pressure ulcers (*P* = .004). Furthermore, the data presented in Table 4 indicate that the average age of patients with pressure ulcers surpasses that of those without, revealing a significant demographic distinction. The aging process induces unavoidable physiological changes in both the body and skin, including intrinsic alterations such as wrinkling, thinning, sagging, and increased fragility. Consequently, elderly patients demonstrate a heightened susceptibility to pressure ulcer development, a phenomenon that aligns with existing research findings.^[13]^

The 2019 update of the Clinical Practice Guideline on the Prevention and Treatment of Pressure Ulcers/Injuries emphasizes that prolonged tissue ischemia, hypoxia, and changes in the microenvironment can reduce local skin tissue tolerance, increasing vulnerability to pressure injury. Therefore, the duration of prone ventilation directly influences the incidence and severity of pressure ulcers. To reduce stress on the skin, it is recommended to intermittently adjust the head position, alternating pressure on the cheeks every 2 to 3 hours, and utilizing circular protective pads.^[14]^ These measures may effectively alleviate local compression and restore blood flow to the affected areas. Our research further confirmed that circular pillows enabling segmental local pressure adjustment can achieve better clinical results.

The 7 inflatable balloons in the modified U-shaped pillow can change the stress points on the U-shaped pillow by adjusting, so as to improve the local blood circulation in the compressed area. Previous research suggests that alternating between higher and lower peak pressures at a single point can enhance cutaneous blood flow more effectively than maintaining a constant low pressure.^[15]^ This mechanistic principle – that cyclical pressure alternation more effectively prevents sustained blood flow obstruction – is corroborated by our findings. The observed reduction in pressure injury incidence in the intervention group aligns with the concept that dynamic pressure redistribution is superior to static support in preventing tissue ischemia. This study showed that the modified U-shaped pillow showed a promising trend in reducing the incidence of pressure injury on the head and face of patients, although there was no statistically significant difference in the severity of pressure injury between the 2 groups. This nonsignificance may be attributed to the increased nursing workload in the control group, since more frequent repositioning may have slowed or prevented the progression of pressure ulcers. In addition, the limited sample size in this study may have been another factor contributing to the lack of significant differences in pressure ulcer severity between the 2 groups.

Pressure ulcers impose a significant burden on both patients and society, making prevention and treatment strategies a central focus of ongoing research.^[16]^ The management costs associated with pressure ulcers are notably high, adversely impacting the delivery of cost-effective and efficient nursing services.^[17]^ The presence of pressure injuries requires nurses to enhance patient care by increasing the frequency of repositioning, administering medications, and changing dressings, thereby significantly augmenting their workload.

This study found that the average frequency of head position adjustments per hour during prone positioning in the control group significantly exceeded that of the study group, indicating a higher nursing workload in the former. In terms of nurse satisfaction, evaluations of operational convenience revealed significantly greater satisfaction levels among nurses in the study group compared to the control group, with a statistically significant difference. In the study group, the use of enhanced U-shaped pillow can directly adjust the inflation level through the air valve, without the need to relocate the patient or adjust the pillow, so that the nurses’ operation efficiency was significantly improved.

Furthermore, the materials used in this design include standard cotton fabric, which offers excellent breathability, strong water absorption, ease of cleaning and disinfection, and effective prevention of cross-infection. The varied materials used for the filling components within the compartments also allow for adaptability to specific clinical scenarios, making this design easy to endorse.

However, this study has several limitations. First, the limited sample size affects its generalizability. Secondly, the inherent biases of retrospective studies may affect the accuracy of the results. Bias in the calculation of statistical power is also inevitable. Future studies should enroll more patients and employ prospective cohort designs to improve the scientific rigor of these findings.

## 5. Conclusion

The application of the original improved U-shaped pillow was associated with a reduced incidence of craniofacial pressure injuries in prone-ventilated patients after adjustment for confounders, while also mitigating the clinical nursing burden and improving job satisfaction among healthcare staff.

## Acknowledgments

Limei Zhu, Xiangwu Huang, Chenxing Jian served as co-corresponding authors with joint responsibility for this study. The authors would like to extend their gratitude to the medical staff who participated in the nursing care of this project. Their dedication and expertise were invaluable to the success of this research. We thank Yingying Jiang for his assistance in the statistical analysis process of this study.

## Author contributions

**Conceptualization:** Xuekui Wang, Lisang Fu, Yanbin Ye, Limei Zhu, Xiangwu Huang, Chenxing Jian.

**Data curation:** Xuekui Wang, Yanbin Ye, Huilin Chen, Yan Wang, Biying Huang, Meifen Chen, Meixian Chen, Limei Zhu.

**Formal analysis:** Xuekui Wang, Lisang Fu, Yanbin Ye, Yan Wang, Biying Huang, Meifen Chen, Meixian Chen, Xiangwu Huang, Chenxing Jian.

**Funding acquisition:** Chenxing Jian.

**Investigation:** Xuekui Wang, Lisang Fu, Huilin Chen, Yan Wang, Biying Huang, Meifen Chen, Meixian Chen, Limei Zhu, Xiangwu Huang, Chenxing Jian.

**Methodology:** Xuekui Wang, Lisang Fu, Huilin Chen, Yan Wang, Chenxing Jian.

**Project administration:** Lisang Fu, Xiangwu Huang, Chenxing Jian.

**Resources:** Chenxing Jian.

**Software:** Yan Wang.

**Supervision:** Limei Zhu, Xiangwu Huang, Chenxing Jian.

**Validation:** Xiangwu Huang, Chenxing Jian.

**Visualization:** Yan Wang.

**Writing – original draft:** Xuekui Wang, Yanbin Ye, Huilin Chen, Yan Wang, Biying Huang, Meifen Chen, Meixian Chen.

**Writing – review & editing:** Xuekui Wang, Lisang Fu, Limei Zhu, Xiangwu Huang, Chenxing Jian.
